# Oral versus intravenous methylprednisolone for the treatment of multiple sclerosis relapses: A meta-analysis of randomized controlled trials

**DOI:** 10.1371/journal.pone.0188644

**Published:** 2017-11-27

**Authors:** Shuo Liu, Xiaoqiang Liu, Shuying Chen, Yingxiu Xiao, Weiduan Zhuang

**Affiliations:** 1 Neurology Department, First Affiliated Hospital of Shantou University Medical College, Shantou, Guangdong, China; 2 Shantou University Medical College, Shantou, Guangdong, China; Karolinska Institutet, SWEDEN

## Abstract

**Background:**

Intravenous glucocorticoids are recommended for multiple sclerosis (MS). However, they can be inconvenient and expensive. Due to their convenience and low cost, oral glucocorticoids may be an alternative treatment. Recently, several studies have shown that there is no difference in efficacy and safety between oral methylprednisolone (oMP) and intravenous methylprednisolone (ivMP).

**Objectives:**

We sought to assess the clinical efficacy, safety and tolerability of oral methylprednisolone versus intravenous methylprednisolone for MS relapses in this meta-analysis.

**Methods:**

Randomized controlled trials (RCTs) evaluating the clinical efficacy, safety and tolerability of oral methylprednisolone versus intravenous methylprednisolone for MS relapses were searched in PubMed, Cochrane Library, Medline, EMBASE and China Biology Medicine until October 25, 2016, without language restrictions. The proportion of patients who had improved by day 28 was chosen as the efficacy outcome. We chose the risk ratio (RR) to analyze each trial with the 95% confidence interval (95% CI). We also used the fixed-effects model (Mantel–Haenszel approach) to calculate the pooled relative effect estimates.

**Results:**

A total of 5 trials were identified, which included 369 patients. The results of our meta-analysis revealed that no significant difference existed in relapse improvement at day 28 between oMP and ivMP (RR 0.96, 95% CI 0.84 to 1.10). No evidence of heterogeneity existed among the trials (P = 0.45, I^2^ = 0%). Both treatments were equally safe and well tolerated except that insomnia was more likely to occur in the oMP group compared to the ivMP group.

**Conclusion:**

Our meta-analysis reveals strong evidence that oMP is not inferior to ivMP in increasing the proportion of patients experiencing clinical improvement at day 28. In addition, both routes of administration are equally well tolerated and safe. These findings suggest that we may be able to replace ivMP with oMP to treat MS relapses.

## Introduction

MS is an inflammatory demyelinating disease that destroys the myelin sheaths of neurons in the central nervous system. Among central nervous disorders, MS is the leading cause of disability in young adults following trauma [1,2]. It is characterized by a varying array of neurological deficits, including challenges with weakness, fatigue, spasticity, gait, cognition, bladder and bowel. Based on the 1996 multiple sclerosis phenotype descriptions, there are four main subtypes of MS: relapsing-remitting(RR), secondary progressive (SP), primary progressive (PP) and progressive relapsing (PR).[3] PR is eliminated in the 2013 revision.[4] Among these subtypes, RRMS is the most common and comprises approximately 85% to 90% of cases at disease onset [5]. It is characterized by periods of exacerbation followed by periods of remission. Most patients with RRMS will eventually enter a secondary progressive phase in which neurological deficits become fixed and accumulate. Glucocorticoids may promote short-term functional recovery in acute MS relapses, and they have been recommended as the first-line treatment for MS relapses [6–8]. Several randomized controlled trials, a systematic review, and a meta-analysis had proven that glucocorticoids can reduce the risk of MS exacerbation compared to placebo in the short term [6,9–11]. However, the route of glucocorticoid administration has not be studied. Furthermore, the use of intravenous glucocorticoids will increase costs, require hospitalization and interfere with daily life while oral glucocorticoids are less expensive, less invasive and more convenient. A systemic review of five RCTs [12–16] comparing oral and intravenous methylprednisolone for MS relapses showed that there is no significant difference in the efficacy of oral and intravenous administration of glucocorticoids [17]. However, the authors of this review showed that there are several limitations of this study including methodological weaknesses, insufficient statistical power, the small number of trials and the small number of participants. They recommended larger trials be conducted. In 2015, a large and adequately powered randomized controlled trial comparing oral versus intravenous methylprednisolone was reported, and the report concluded that oral methylprednisolone was not inferior to intravenous methylprednisolone in efficacy [18]. Thus, the aim of our meta-analysis was to compare the efficacy and safety of oral and intravenous methylprednisolone.

## Methods

### Search methods

The Preferred Reporting Items for Systematic reviews and Meta-Analysis (PRISMA) checklist is shown in the **S1 Checklist**. According to PRISMA, a detailed protocol was developed prior to this study (dx.doi.org/10.17504/protocols.io.j5hcq36 [PROTOCOL DOI]). Five databases were used including PubMed, EMBASE, Cochrane Library, Medline and China Biology Medicine from the start of the study to October 25, 2016. Two researchers searched these electric databases independently. Keywords including multiple sclerosis, oral, intravenous, glucocorticoid, methylprednisolone and Randomized Controlled Trials were used. There were no language limitations for inclusion. After removing duplicated reports and unrelated articles, we conducted the meta-analysis by using the remaining articles.

### Inclusions/exclusions

Inclusion criteria included the following: (1) participants ≥16 years old and who were diagnosed with MS with an acute relapse event; (2) studies comparing oral methylprednisolone with intravenous methylprednisolone directly without restrictions on the dosage used; (3) outcomes regarding clinical efficacy; (4) the use of Kurtzke’s EDSS (expanded disability status scale) or a functional system of Kurtzke’s EDSS to measure outcomes of disability within six weeks after treatment; and (5) RCTs. A trial was sometimes broken down into several articles. We either integrated these different articles of the same trial into one study or chose the article with the most detailed information and complete data. Titles and abstracts were reviewed to eliminate irrelevant articles. Potential papers were evaluated by reading the full text.

### Data extract and quality assessment

Two reviewers independently extracted the data from the included trials. Any inconsistency was resolved by consultation until a consensus was reached. Details of the following variables were extracted: 1) baseline characteristics of participants; 2) interventions (drug, route, dosage and duration) and duration of follow up in each group; 3) positive events of primary outcomes and the total numbers of participants in each group. We assessed the quality of the methods by evaluating all potential problems that would have resulted in a bias, including random sequence generation; allocation concealment; blinding of participants and personnel; blinding of outcome assessment; incomplete outcome data reporting; selective reporting and other biases. The risk of each bias was classified as low risk, unclear risk (insufficient information) or high risk. We used this classification to grade the bias in each domain. [19]

### Definition of outcomes

We only chose the same efficacy outcome that was reported with detailed data that was available in the five studies that were included as part of the meta-analysis as our efficacy outcome. Furthermore, the proportion of patients with improvement in MS relapse after glucocorticoid treatment at four weeks was chosen as our efficacy outcome. Safety and tolerability outcomes included any serious adverse events and common adverse events including rash, anxiety, insomnia, dysgeusia, stomach pain, headache, euphoria, nausea, diarrhea and palpitations. Adverse events were noted at the follow-up duration.

### Data synthesis and analysis

The proportion of patients with relapse improvement after treatment and adverse events were represented as dichotomous data. We chose the RR to analyze each trial with the 95% CI, and a P value <0.05 was defined as statistically significant. We calculated the Cochran’s Q value and I^2^ statistic to determine the heterogeneity of all trials that were included in the analysis [20]. A Q statistic < 0.05 indicates substantial heterogeneity. An I^2^ value <40% is defined as not exhibiting heterogeneity while I^2^>75% indicates considerable heterogeneity. I^2^ values ranging from 30% to 60% represent moderate heterogeneity while those values ranging from 50% to 90% represent substantial heterogeneity [19].

In the absence of heterogeneity, we used the fixed-effects model (Mantel–Haenszel approach) to calculate the pooled relative effect estimates. Otherwise, if heterogeneity existed, we performed a sensitivity analysis to achieve homogeneity and used the fixed-effects model. If heterogeneity still existed after the sensitivity analysis, we chose the random effects model (the DerSimonian-Laird estimator and the Mantel–Haenszel approach) to conduct this meta-analysis. Publication bias was evaluated by using the funnel plot and the Egger’s test [21]. For all statistical analyses, we used Review Manager 5.3 and Stata 11.

## Results

### Search result

**Fig 1:**
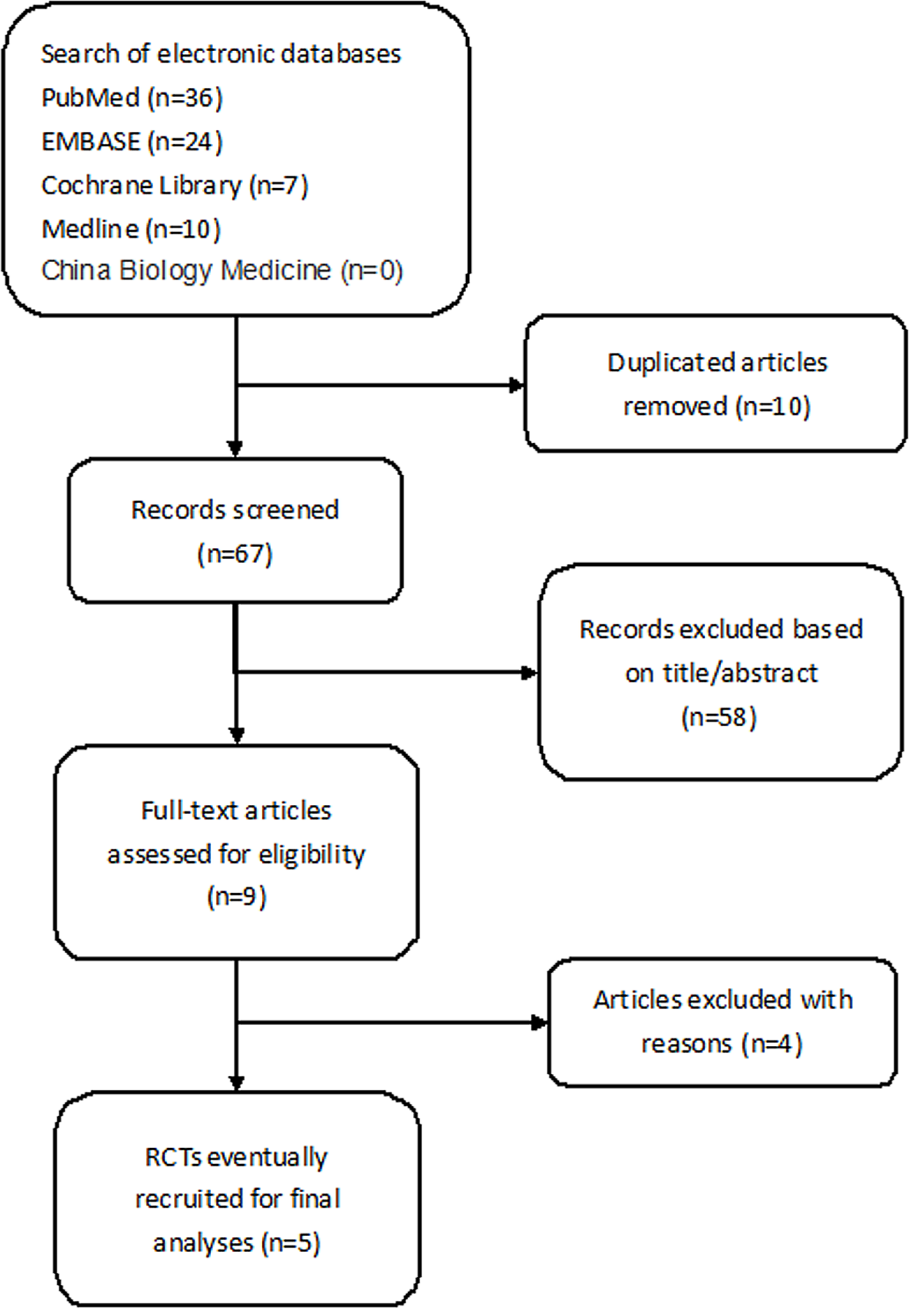
PRISMA flow diagram.

Our electronic search identified 77 articles; 10 of them were duplicated and were excluded. Among the remaining articles, 58 articles were excluded based on titles and abstracts, as the participants and interventions in these studies were irrelevant to the aim of this meta-analysis. Finally, the remaining 9 studies were potentially eligible articles [12–16,18,22–24]. After reviewing the full article, we found that three of them were based on one trial [13,22,23]. Therefore, two of them were excluded [22,23]. Two other articles were based on the same trial [15,24] and, again, one of them was rejected [24]. One article only compared the bioavailability of glucocorticoids between oral and intravenous administration but not clinical efficacy; therefore, we discarded this study as well [16]. Eventually, 5 RCTs comparing the clinical efficacy, safety and tolerability of oral methylprednisolone (oMP) vs intravenous methylprednisolone (ivMP) for MS were further analyzed [12–15,18]. **Fig 1** shows the flow diagram.

### Characteristic of studies

**Table 1** presents summaries of the study characteristics. Among the five RCTs, there were 398 randomized patients in total, with 198 patients in the oral glucocorticoid group and 200 patients in the intravenous group. Study sample sizes ranged from 35 to 199. In each study, the mean or median age among the oMP and ivMP groups were similar, ranging from 31 years old to 41.6 years old. For the intervention, all five studies compared oMP and ivMP. Two of the studies used identical doses (1000 mg/d), with one study administering methylprednisolone for 3 days while the other administered it for 5 days. One study used a bioequivalent dose (1250 mg/d of oMP vs 1000 mg/d of ivMP) for 3 days. Furthermore, another study used 500 mg/d of methylprednisolone for 5 days. The last study used a much lower dose but had a longer duration regimen (48 mg/d of oMP for 7 d, then 24 mg/d for 7 d, and 12 mg/d for 7 d versus the conventional 1000 mg of ivMP). All studies examined the same clinical outcome, which was the proportion of patients with improvement at day 28, to assess efficacy. Four of the studies used Kurtzke’s EDSS and one used the functional system of Kurtzke’s EDSS to evaluate the clinical outcome. The duration of follow up ranged from 4 to 24 weeks.

**Table 1:** Characteristics of studies.

study	number of patients (oMP/ivMP)	mean/median age, years (oMP/ivMP)	number of women (oMP/ivMP)	duration of disease, years (oMP/ivMP)	drug, route, dosage and duration		duration of follow up	inclusion criteria
					oMP	ivMP		
Alam 1993	38(18/20)	41.3/41.6	11/16	3.8/6.5	500mg/d of MP for 5 days	500mg/d of MP for 5 days	4 weeks	1. clinically definite MS; 2.symptomatic deterioration within 4 weeks
Barnes 1997	80(42/38)	38/37	24/27	6.6/6.3	48mg/d of MP for 7d, 24mg/d for 7d, 12mg/d for 7d	1000mg/d of MP for 3 days	24 weeks	1. clinically definite MS; 2. symptomatic deterioration within 4 weeks
Martinelli 2008	40(20/20)	36/31	13/14	9.8/7.2	500mg of MP BID for 5 days	1000mg/d of MP for 5 days	4 weeks	1. relapsing-remitting MS; 2. in relapse phase
Ramo-Tello 2013	49(25/24)	39.5/37.7	17/19	none	1250mg/d of MP for 3 days	1000mg/d of MP for 3 days	12 weeks	1. relapsing-remitting MS; 2. symptomatic deterioration within 15 days
Emmanuelle Le Page 2015	199(100/99)	35/ 34.7	74/74	6.2/5.7	1000mg/d of MP for 3 days	1000mg/d of MP for 3 days	24 weeks	1. relapsing-remitting MS; 2.symptomatic deterioration within 15 days

### Risk of bias

**Fig 2:**
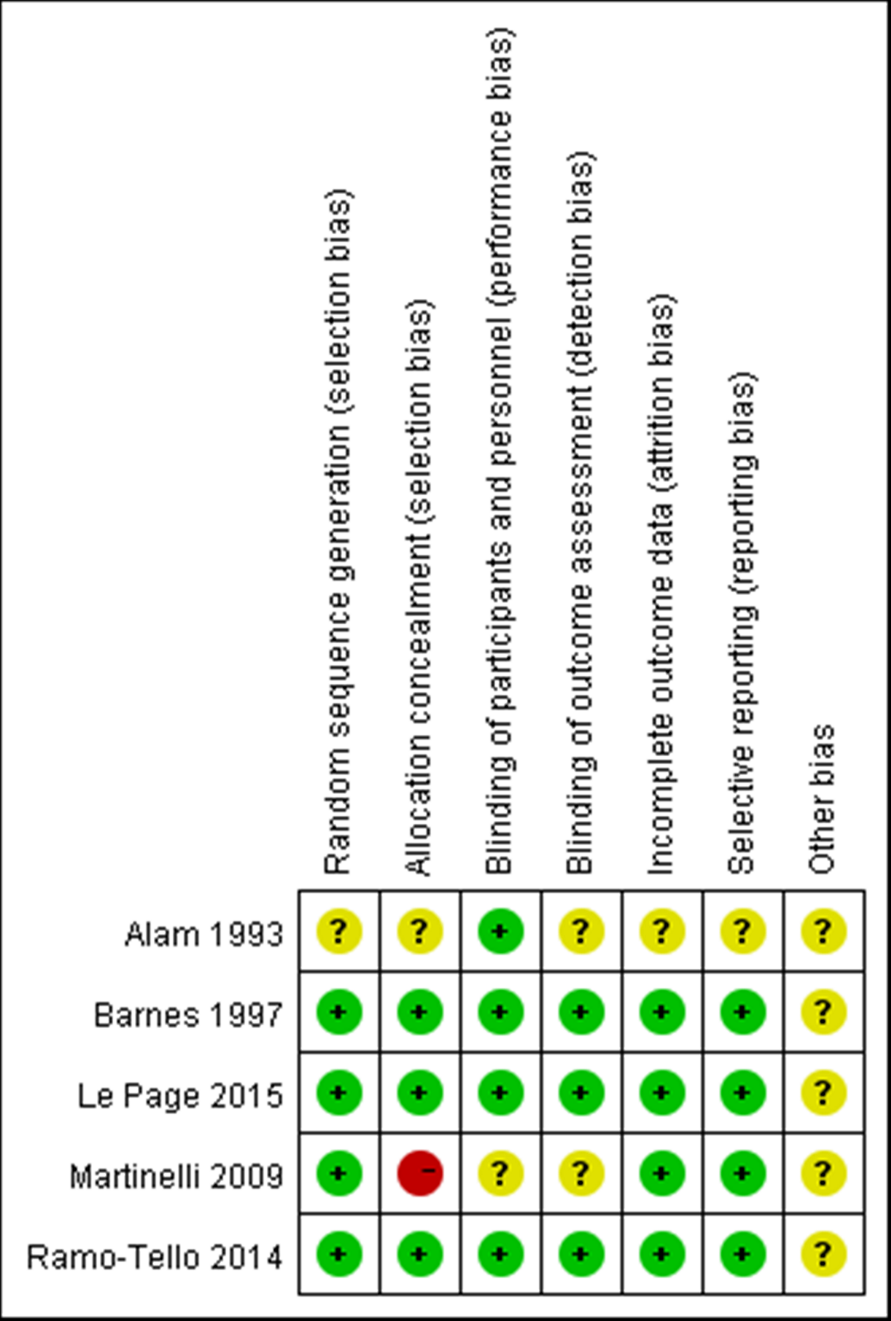
Risk of bias. Green indicates low risk; yellow indicates unclear risk; red indicates high risk.

The methodological quality of the 5 RCTs was independently evaluated by two review authors, and any disagreements were resolved after discussion. Among the 5 studies, 3 of them generated a random sequence; concealed allocation; blinded participants; personnel and outcome assessment; issued incomplete outcome data and addressed selective reporting [13,15,18]. One of the studies only reported that they generated a random sequence; blinded outcome assessment and addressed selective reporting but did not conceal allocation [14]. Other methods were unclear. The last study simply mentioned the blinding of participants and personnel [12]. Details are shown in **Fig 2,** which proved that our meta-analysis was based on studies with a low risk of bias and would provide strong evidence for making clinical decisions.

### Results of meta-analyses

#### Efficacy outcome

**Fig 3:**
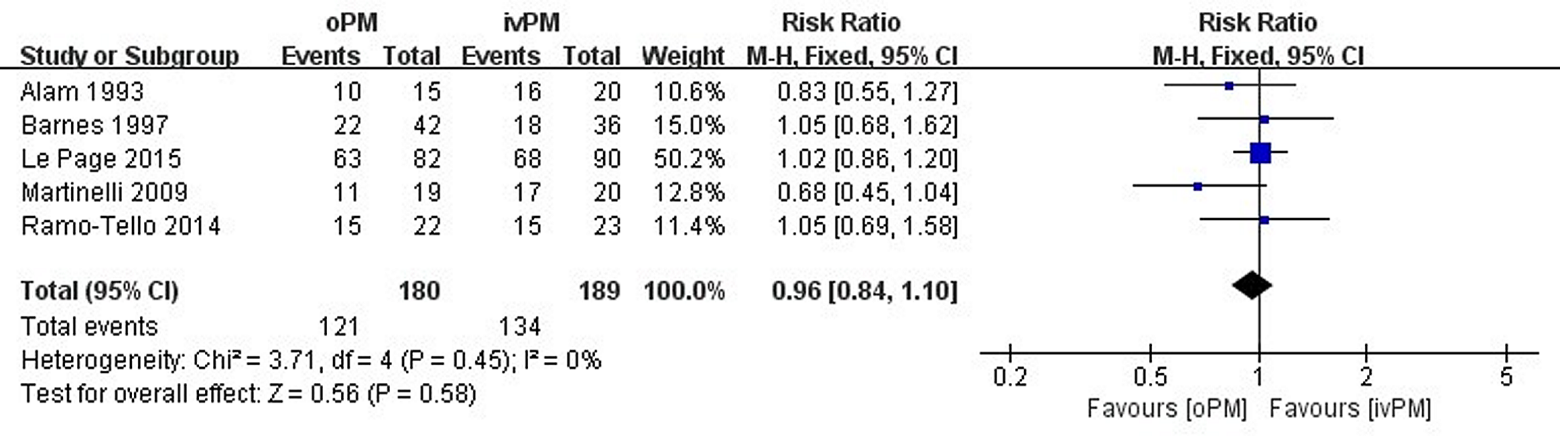
Forest plot.

All of the studies that were included reported the proportion of patients experiencing improvement with oMP vs ivMP treatment at four weeks. Every trial reported that there was no significant difference in the oMP group compared to the ivMP group. We chose the Mantel-Haenszel method and the fixed effect model to conduct a pooled analysis of efficacy. There were 121 improvement events in 180 participants that occurred in the oMP group vs 134 improvement events in 189 participants in the ivMP group. The RR was 0.96 (95% CI 0.84 to 1.10), which is not statistically significant. The P value from the Cochran’s Q test was 0.45, and the corresponding I^2^ was 0%. They both implied little heterogeneity among these studies. **Fig 3** is the forest plot of the efficacy outcome.

#### Publication bias

**Fig 4:**
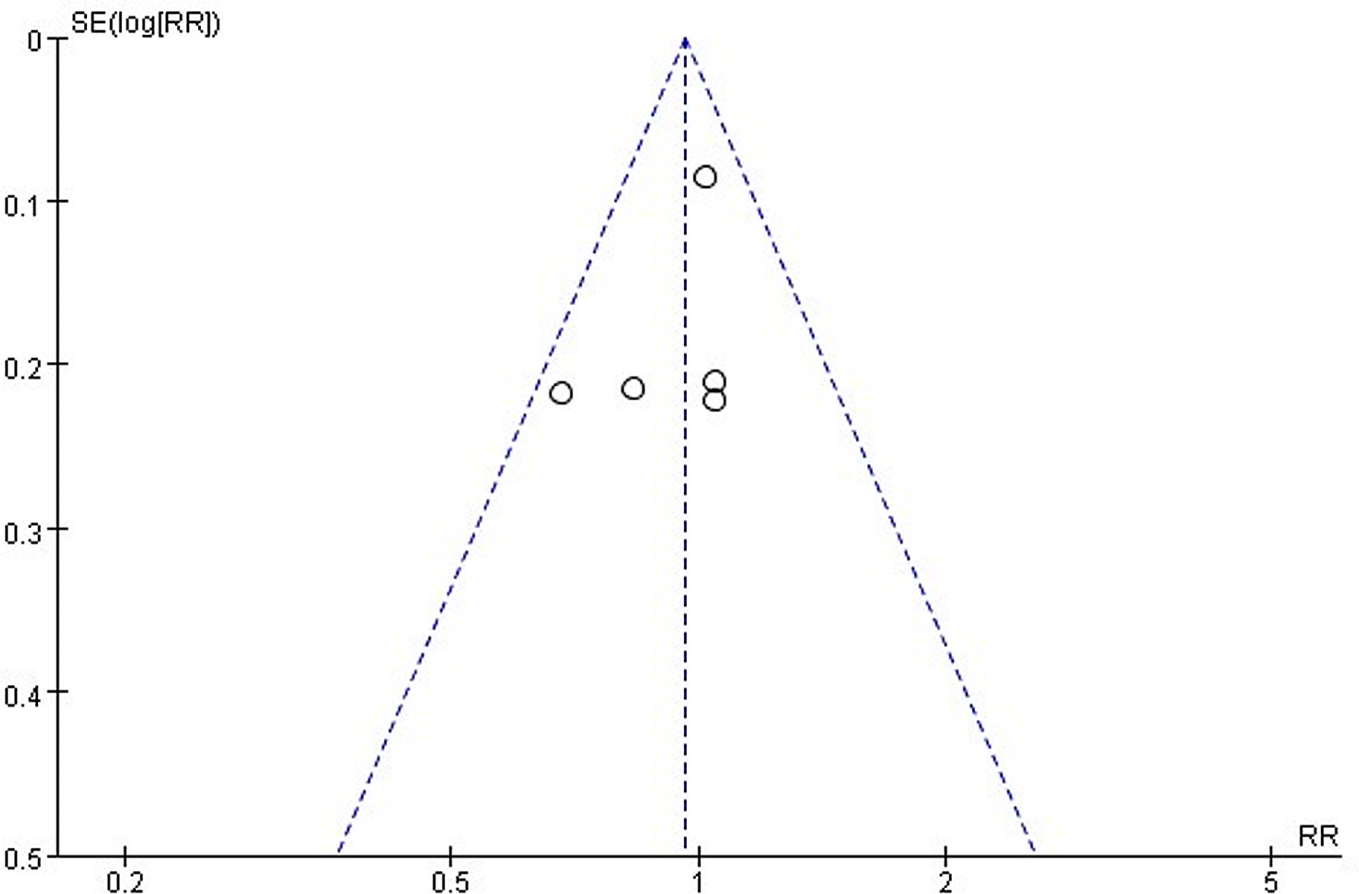
Funnel plot.

A funnel plot **(Fig 4)** showed a nearly symmetrical distribution of the efficacy outcomes of the studies. The P value for the Egger’s test was 0.385, and the 95% confidence interval was -3.64 to 1.88. All analyses revealed that the efficacy outcomes of the studies did not indicate significant publication bias. However, considering the relatively small numbers of trials that were included, the results of the Egger’s test and funnel plot may be influenced by a type II error, which depends on the power of the test.

#### Adverse events

**Fig 5:**
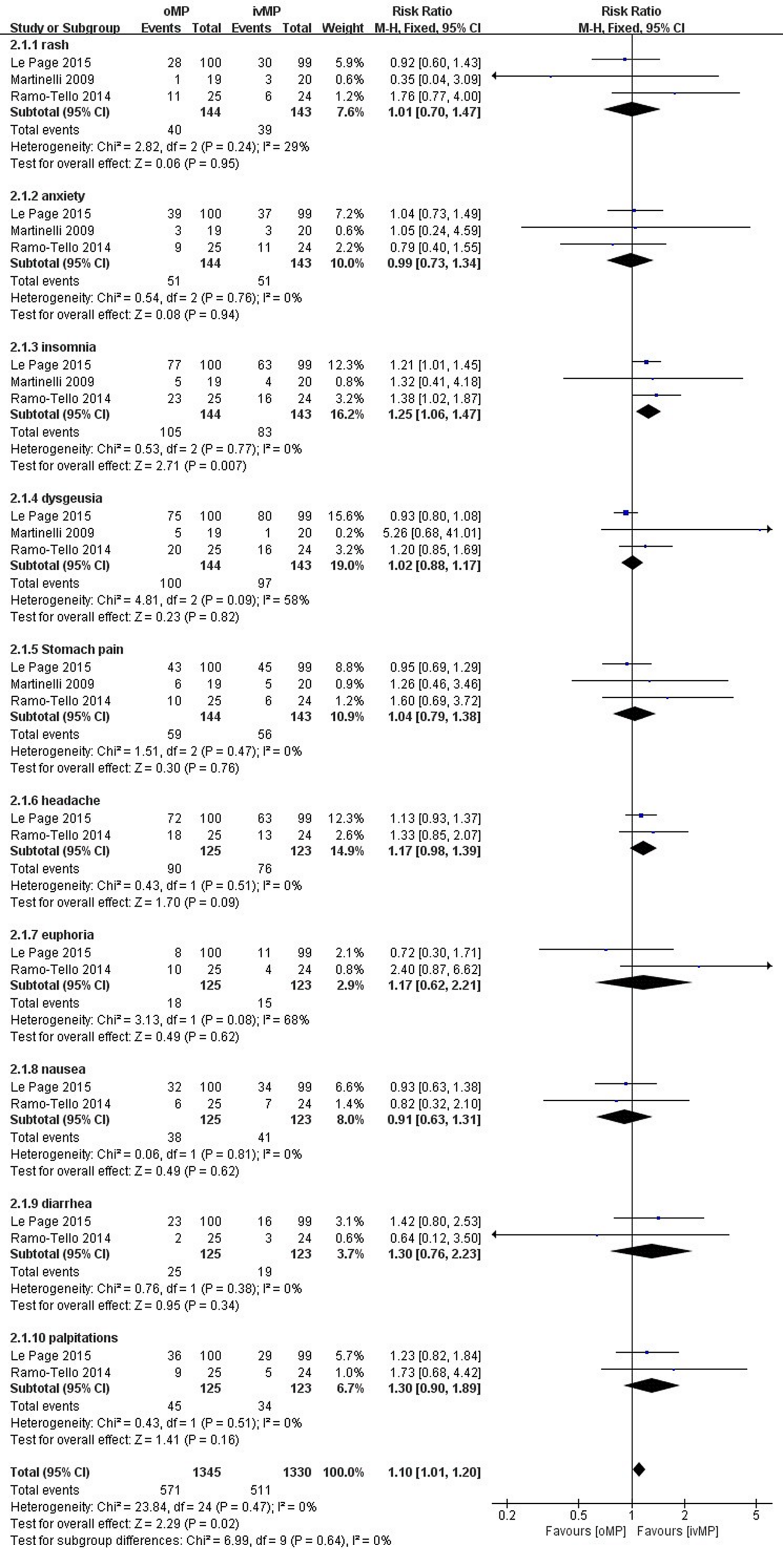
Adverse effects.

Three of the five studies that were included reported several of the same adverse events including rash, anxiety, insomnia, dysgeusia, stomach pain, headache, euphoria, nausea, diarrhea and palpitations. However, an additional two studies only concluded that there were no severe adverse effects in either group, and one of the studies reported no significant difference in adverse events such as headaches, acne, ankle edema, indigestion, a sensation of feeling flushed, dizziness, phlebitis and mild depression between both groups without any detailed data [12,13]. Therefore, we excluded these two studies to perform subgroup analyses of adverse events **(Fig 5)**. In addition, our results showed that no significant difference existed between study groups in the rates of rash (RR 1.01, 95% CI 0.70 to 1.47), anxiety (RR 0.99, 95% CI 0.73 to 1.34), dysgeusia (RR 1.06, 95% CI 0.98 to 1.14), stomach pain (RR 1.04, 95% CI 0.79 to 1.38), headache (RR 1.17, 95% CI 0.98 to 1.39), euphoria (RR 1.17, 95% CI 0.62 to 2.21), nausea (RR 0.91, 95% CI 0.63 to 1.31), diarrhea (RR 1.30, 95% CI 0.76 to 2.23) and palpitations (RR 1.30, 95% CI 0.90 to 1.89). However, insomnia was more likely to occur in the oMP group than the ivMP group (RR 1.25, 95% CI 1.06 to 1.47). Hypertension, hypertrichosis, hiccups, hyperglycemia, mood disturbance, hot flashes, edema, fatigue, agitation, vomiting and chest pain were reported in one study, and no significant difference existed between the oMP and ivMP groups. None of the reported adverse effects were serious.

## Discussion

Glucocorticoids are currently recommended for the treatment of MS relapses. However, the route of drug administration has not been established. Therefore, we conducted a meta-analysis to compare the clinical efficacy, safety and tolerability of glucocorticoids *via* two routes, oMP vs ivMP. By October 25, 2016, only 5 RCTs comparing the clinical efficacy, safety and tolerability between oMP and ivMP had been reported. Two of these trials used identical doses (1000 mg/d). One used a bioequivalent dose (1250 mg/d of oMP vs 1000 mg/d of ivMP), and another used 500 mg/d of methylprednisolone. The last trial used a much lower dose but had a longer duration regimen (48 mg/d of oMP for 7 d, then 24 mg/d for 7 d, and 12 mg/d for 7 d versus the conventional 1000 mg of ivMP). All five studies concluded that, compared to ivMP, oMP was not inferior in the proportion of patients who improved at 28 days after a relapse. Of the studies that were included, only three studies reported adverse event rates. Our meta-analysis shows that oral and intravenous methylprednisolone are equally well tolerated and safe except that insomnia was more likely to occur in the oMP group than in the ivMP group. This adverse effect might be due to the prolonged bioavailability of oMP [18]. Therefore, in order to avoid insomnia, we recommend taking oMP in the morning. A systematic review written by Burton [17] comparing oral versus intravenous methylprednisolone also revealed that there was no statistically significant difference between the two routes of steroid administration in the proportion of patients who improved at 4 weeks, a finding that is consistent with our efficacy outcome. In addition, this study reported that oMP and ivMP were equally safe and well tolerated except a tendency towards more cases of dysgeusia and mood disturbance with oral steroid administration. Our study also revealed that both routes were equally safe and well tolerated, except for an increased incidence of insomnia but not dysgeusia in the oMP group. The findings are slightly different from what was found in the Cochrane systematic review. We believe our findings are more reliable because we included more trials and numbers of participants to analyze the outcome of adverse effects than in the previous systematic review. For mood disturbance, we did not analyze this factor because only one study had mentioned it.

The systematic review written by Burton addressed several limitations including the small number of trials, the small number of participants, design heterogeneity and methodological limitations. They indicated that more large scale and better designed trials needed to be done. Our meta-analysis included four of the same trials that were included in Burton’s systematic review, but we excluded the last study because it compared the bioavailability of oral and intravenous glucocorticoids but did not examine their clinical efficacy. We also included a large, adequately powered RCT comparing oMP versus ivMP in MS, which was reported in 2015 [18]. In addition, we only combined the same evaluation target of the five studies into our primary efficacy outcome. By doing so, we reduced the amount of bias and error and increased the statistical power compared with the previous Cochrane systematic review.

In our study, although proper statistic methods were used and little heterogeneity was detected among the five studies that were included, certain limitations must be addressed. First, the dose of the MP in each study was not identical. One study used a bioequivalent dose, three used identical doses and one used neither bioequivalent nor identical doses. Second, several studies enrolled participants within one month following a relapse. Treatment could be delayed because participants may enter the resolution phase of a relapse within a month. Third, several studies lacked reliable randomization methods, blinding methods, allocation concealment or appropriate assessment. Finally, the five studies that were included only reported a single primary outcome with detailed data, so there is only one efficacy outcome in our meta-analysis. Specifically, more RCTs should be conducted on a large scale with sufficient power to compare oMP versus ivMP in MS relapse.

In this study, we show that oMP was not inferior to ivMP in improvement of disability scores 28 days after a relapse. There are similar studies comparing oral corticosteroids with intravenous corticosteroids in treating acute exacerbation of chronic obstructive pulmonary disease (AECOPD) and asthma. Several studies examining the efficacy of oral versus intravenous corticosteroids in the treatment of AECOPD showed no significant difference in the primary outcomes of treatment failure, relapse or mortality.[25–28] And current guidelines for the treatment of AECOPD recommend oral corticosteroids for the exacerbations.[26,29–31] Studies comparing oral with intravenous corticosteroids in the acute exacerbation of asthma also support the use of oral corticosteroids.[32–35] The result of our study agreed with the experience in the field of AECOPD and asthma. This result may be explained by the similar bioavailability of oral with intravenous corticosteroids. A small trail conducted by Marrow had suggested that a very high dose of oral prednisone is safe and has similar bioavailability to ivMP.[16] Considering the efficacy, safety, good bioavailability,[36] lower costs and greater ease of administration, oMP for MS relapse events may be a reasonable treatment alternative to ivMP.

## Conclusion

In conclusion, despite several limitations, our meta-analysis revealed that there are no significant differences in clinical efficacy and adverse events between oral and intravenous methylprednisolone for the treatment of relapses in multiple sclerosis. Based on the evidence, oral steroids, which are less expensive, less invasive and more convenient, may be an effective alternative to intravenous steroids for the treatment of MS relapses.

## Supporting information

S1 Checklist PRISMA checklist. (DOC)

S1 Text Manuscript protocol. (DOC)

S2 Text Summary of literature search and selection process. (DOCX)
