# Supplementary material for: Oral versus intravenous methylprednisolone for the treatment of multiple sclerosis relapses: A meta-analysis of randomized controlled trials
Source: PLoS One. 2017 Nov 27;12(11):e0188644. doi: 10.1371/journal.pone.0188644 (PMC5703548; doi:10.1371/journal.pone.0188644)
Supplement: S1 Text — (DOC) [file pone.0188644.s002.doc]

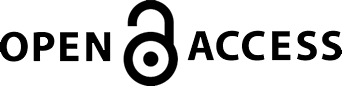

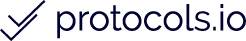
**Oral versus intravenous methylprednisolone for treatment of relapses in multiple sclerosis：a meta-analysis of randomized controlled trials.**

**
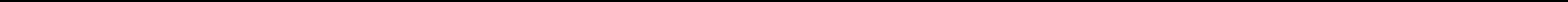
**

**Shuo Liu**

**
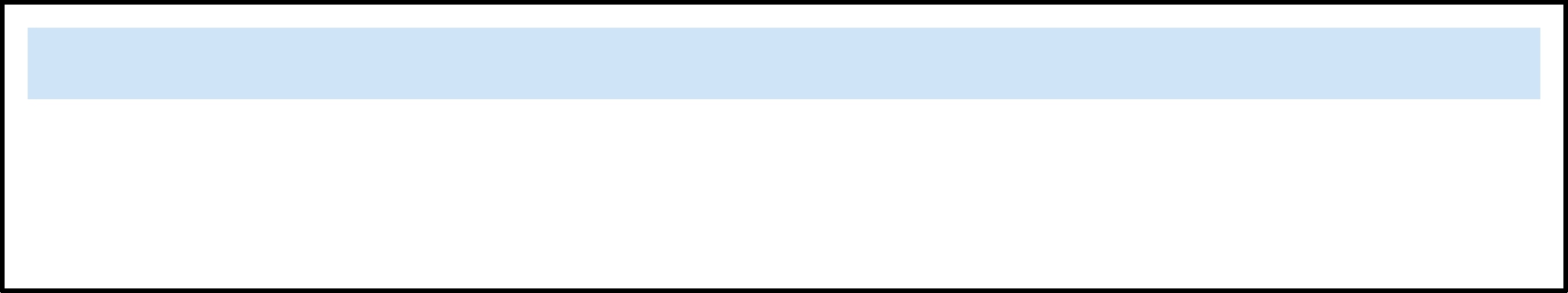
**

**Abstract**

**Citation:** Shuo LiuOral versus intravenous methylprednisolone for treatment of relapses in multiple sclerosis：a meta-analysis of randomized controlled trials.. **protocols.io**

https://www.protocols.io/view/oral-versus-intravenous-methylprednisolone-for-tre-j5hcq36

**Published:** 01 Oct 2017

**Protocol**

**
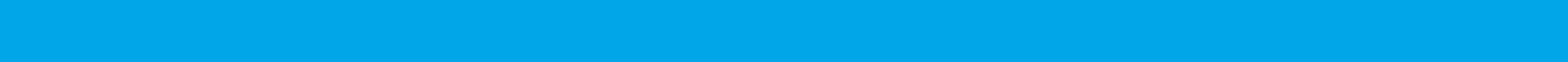
**

Background

1. Multiple sclerosis is an inflammatory demyelinating disease，which destroys myelin sheaths of neurons of central nervous system. Glucocorticoid has been recommended as the first line treatment for relapses of MS. However, route of giving glucocorticoid has not be determined. A systemic review of five RCTs comparing oral and intravenous methylprednisolone for relapses of MS showed that there is no significant diﬀerences in eﬃcacy between oral and intravenous administration. But the authors of this review indicated that there are some limitations of this study such as methodological weaknesses, insuﬃcient statistical power, small trial number and small number of participants. They called for more large scale trials to be done. And in 2015, a large, adequately powered, randomized controlled trial comparing oral versus intravenous methylprednisolone was reported.

**
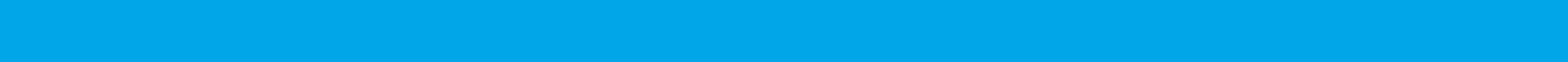

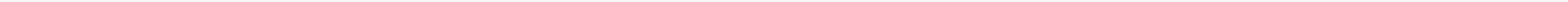
**

Why it is important to do this review

1. Intravenous glucocorticoid will increase cost, need hospitalization and influence daily life while oral glucocorticoid is cheaper, less invasive and more convenient. So we do this meta-analysis to find out whether oral glucocorticoid can be an eﬀective alternative to intravenous steroids for MS relapse.

**
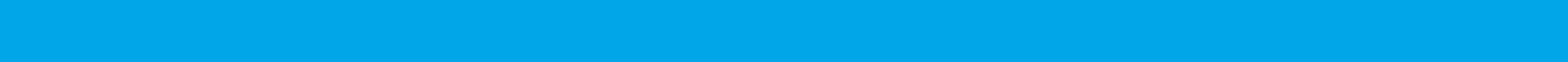

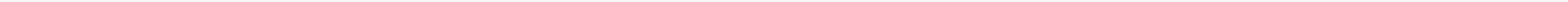
**

Objectives

1. To compare eﬃcacy and safety between oral and intravenous methylprednisolone.

.


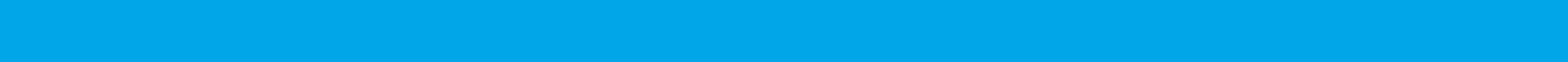

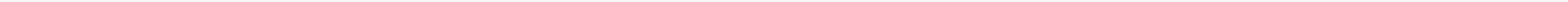


Design

**4.** Traditional meta analysis.


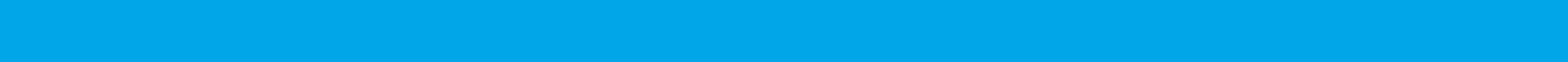

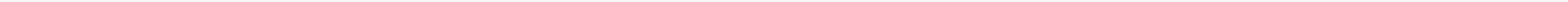


Data Source

**5.** PubMed, EMBASE, Cochrane Library, MEDLINE and China Biology Medicine.


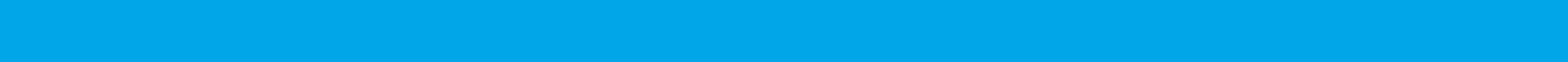

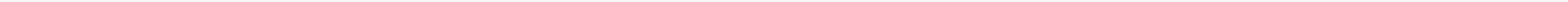


Methods

1. Randomized controlled trials of oral glucocorticoid compared with

intravenous methylprednisolone for multiple sclerosis. Two reviewers independently checked the quality of RCTs. Another two investigators independently extracted data. The primary eﬃcacy outcomes (proportion of patients experiencing improvement in oral methylprednisolone vs

intravenous methylprednisolone at four weeks.) and adverse events were summarized for meta analysis. Stata software was used for traditional meta analysis.


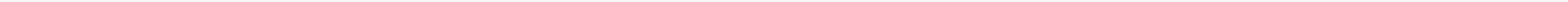


1 **Published:** 01 Oct 2017


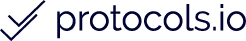


This is an open access protocol distributed under the terms of the [Creative Commons Attribution License](https://creativecommons.org/licenses/by/4.0/), which permits unrestricted use, distribution, and reproduction in any medium, provided the original author and source are credited


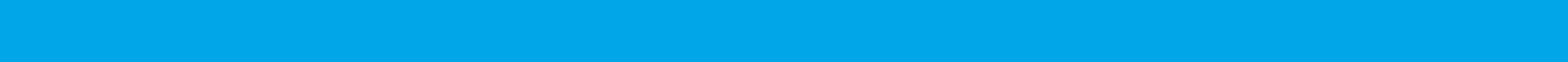
Results

1. A total of 5 trials were identified, including 369 patients. The results of our meta-analysis revealed that no significant diﬀerence existed in relapse improvement at day 28 between oMP and ivMP (risk ratio (RR) 0.96, 95% confidence interval (95% CI) 0.84 to 1.10). No evidence of heterogeneity appeared

among the trials (P=0.45, I^2^ =0%). Both treatments were equally safe and well tolerated except that insomnia was more likely to occur in oMP group than ivMP group.


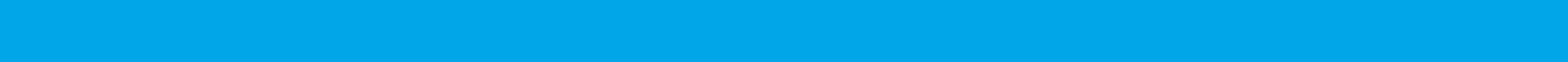

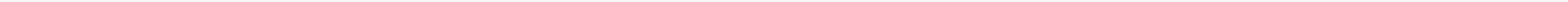


Conclusion

1. Our meta-analysis furnishes strong evidence that oMP is noninferior to ivMP in increasing proportion of patients experiencing improvement at day 28. And both routes of administration are equally well tolerated and safe. This finding suggests that we may replace ivMP with oMP to treat MS relapses.

**
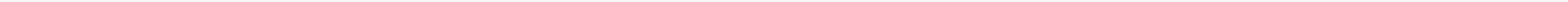
**

2 **Published:** 01 Oct 2017


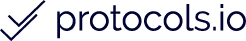


This is an open access protocol distributed under the terms of the [Creative Commons Attribution License](https://creativecommons.org/licenses/by/4.0/), which permits unrestricted use, distribution, and reproduction in any medium, provided the original author and source are credited
