# Supplementary material for: Oral versus intravenous methylprednisolone for the treatment of multiple sclerosis relapses: A meta-analysis of randomized controlled trials
Source: PLoS One. 2017 Nov 27;12(11):e0188644. doi: 10.1371/journal.pone.0188644 (PMC5703548; doi:10.1371/journal.pone.0188644)
Supplement: S2 Text — (DOCX) [file pone.0188644.s003.docx]

Search criteria for Pubmed

(((((("Multiple Sclerosis"[Majr]) OR multiple sclerosis)) AND (((("Glucocorticoids"[Majr]) OR glucocorticoids) OR "Methylprednisolone"[Majr]) OR Methylprednisolone)) AND oral) AND intravenous) AND (((Randomized Controlled Trial) OR "Randomized Controlled Trials as Topic"[Mesh]) OR "Randomized Controlled Trial"[Publication Type])
